# Polymorphisms in *NFkB, PXR*, *LXR *and risk of colorectal cancer in a prospective study of Danes

**DOI:** 10.1186/1471-2407-10-484

**Published:** 2010-09-13

**Authors:** Vibeke Andersen, Jane Christensen, Kim Overvad, Anne Tjønneland, Ulla Vogel

**Affiliations:** 1 Medical Department, Viborg Regional Hospital, Heibergs Allé 4, DK-8800 Viborg, Denmark; 2 Danish Cancer Society, Institute of Cancer Epidemiology, DK-2100 Copenhagen, Denmark; 3 Department of Epidemiology, School of Public Health, Aarhus University, Aarhus, Denmark; 4 National Research Centre for the Working Environment, DK-2100 Copenhagen, Denmark; 5 National Food Institute, Technical University of Denmark, DK-2860 Soborg, Denmark; 6 Institute of Science, Systems and Models, University of Roskilde, DK-4000 Roskilde, Denmark

## Abstract

**Background:**

Transcription factors and nuclear receptors constitute a link between exposure to heterocyclic amines and polycyclic aromatic hydrocarbons from meat and tobacco smoke and colorectal cancer (CRC) risk. The aim of this study was to investigate if polymorphisms in nuclear factor kappa-B, pregnane X receptor, and liver X receptor were associated with risk of CRC, and to investigate possible interactions with lifestyle factors such as smoking, meat consumption, and NSAID use.

**Methods:**

The polymorphisms nuclear factor kappa-B (*NFkB, NFKB1) *-94 insertion/deletion ATTG (rs28362491), pregnane X receptor (*PXR, NR1I2) *A-24381C (rs1523127), C8055T (rs2276707), A7635G (rs6785049), liver X receptor (*LXR-β, NR1H3) *C-rs1405655T, T-rs2695121C were assessed together with lifestyle factors in a nested case-cohort study of 378 CRC cases and 756 random participants from the Danish prospective Diet, Cancer and Health study of 57,053 persons.

**Results:**

Carriers of *NFkB *-94deletion were at 1.45-fold higher risk of CRC than homozygous carriers of the insertion allele (incidence rate ratio (IRR) = 1.45, 95% confidence interval (95% CI): 1.10-1.92). There was interaction between this polymorphism and intake of red and processed meat in relation to CRC risk. Carriers of *NFkB *-94deletion were at 3% increased risk pr 25 gram meat per day (95% CI: 0.98-1.09) whereas homozygous carriers of the insertion were not at increased risk (p for interaction = 0.03). *PXR *and *LXR *polymorphisms were not associated with CRC risk. There was no interaction between use of nonsteroid antiinflammatory drugs (NSAID) or smoking status and *NFkB*, *PXR *or *LXR *polymorphisms.

**Conclusions:**

A polymorphism in *NFkB *was associated with CRC risk and there was interaction between this polymorphism and meat intake in relation to CRC risk. This study suggests a role for NFkB in CRC aetiology.

## Background

Colorectal cancer (CRC) constitutes the second most common cause of all cancer-related incidence and mortality in the Western World [1]. Risk factors include diet, lifestyle factors and genetic predisposition and as much as 50% of the CRC etiology has been attributed to diet [1,2]. Intake of red and processed meat is a risk factor for CRC and Danes have the highest meat intake per capita in the World [1,3,4]. The exact mechanisms by which intake of meat and processed meat promotes carcinogenesis is not clear [5]. Red and processed meat represent sources of carcinogenic heterocyclic amines (HCA), polycyclic aromatic hydrocarbons (PAH) as well as N-nitroso compounds caused by cooking at high temperature and by processing of meat [5]. Moreover, heme in meat may cause oxidative stress, oxidative DNA damage and carcinogenesis [6]. Tobacco smoking has also been reported to confer risk of CRC [7,8] and Danes have a high prevalence of smokers [9,10]. Tobacco contains a large number of mutagenic and carcinogenic compounds, including PAH, nitrosamines, and nicotine [11]. PAHs and other carcinogens from tobacco smoke may reach the intestinal lumen after swallowing of inhaled particles and smoke carcinogens may then be taken up the same way as meat carcinogens. Long term use of aspirin and other non-steroidal anti-inflammatory drugs (NSAID) has been found to confer protection against CRC [12,13]. Important mechanisms may be induction of apoptosis in CRC cells by a NFkB dependent pathway as well as suppression of inflammation [14,15].

Gene-environment interaction studies have provided novel insights into the pathogenesis of CRC [16-19]. We have recently found interaction between meat intake and polymorphisms in the ATP-binding cassette (ABC) transporter B1 *(ABCB1, MDR1) *which encodes glycoprotein P involved in reverse transport of carcinogens and other xenobiotic substances [20]. The expression of human *MDR1 *is regulated by pregnane X receptor (PXR) and nuclear factor-κB (NFkB) [21] whereas other xenobiotic transporters such as *ABCC2 *(*MRP2) *seem to be regulated by PXR and Liver X receptor (LXR) and *ABCG2 *(*BCRP) *by Peroxisome Proliferator-Activated Receptor γ (PPARγ) [21-24].

In relation to cancer development, the most important effects of *NFkB *activation by tobacco smoke, PAH and bacterial components is considered to be inhibition of cell apoptosis caused by the increased transcription of genes such as interleukin 1-β (IL-1β) and cyclooxygenase-2 (COX-2) [25,26]. Such a role for NFkB in tumor promotion is supported by the lower tumor incidence and size of tumors after inactivation of NFkB in animal studies [27]. On the other hand, functional NFkB seem to be required to control inflammation [28,29] and IL-1β expression [30] and to induce apoptosis [15]. A functional ATTG insertion/deletion (ins/del) polymorphism in the promoter region of *NFkB *gene was associated with lower transcription levels of the del-allele in luciferase reporter systems [31] and risk of CRC in a Swedish, but not in a Chinese study population [32].

Activation of *PXR *by xenobiotica, including carcinogens, leads to up-regulation of enzymes and transporters involved in carcinogen handling including *MDR1 *[33] and to repression of *NFkB *and other pro-carcinogenic genes [34]. Persons homozygous for the *PXR *A7635G (rs2276707) G-allele and persons carrying at least one *PXR *C8055T (rs6785049) T-allele have significant higher levels of intestinal *CYP3A *level following *in vivo *treatment with the inducer rifampin than the homozygous wildtype carriers [35]. Activation of *LXR *by fatty acids, bacteria and cytokines leads to repression of pro-carcinogenic genes as *IL-1β, COX-2*, *MMP-9 *and *NFkB *[36]. The *LXR-β *marker polymorphisms rs1405655 and rs2695121 have been investigated as risk genes for Alzheimers disease with negative results [37].

We hypothesized that carriers of the *NFκB *-94del allele and *PXR *A7635G A-allele and C8055T C-allele would be at higher risk of CRC, especially among smokers and with high intake of red meat, whereas a protective effect of nonsteroid antiinflammatory drugs (NSAID) use was expected. We investigated the association between polymorphisms in *NFkB *(NFkB1) -94 insertion/deletion ATTG (rs28362491), *PXR *(NR1I2) A-rs1523127-C, C-rs2276707-T, A-rs6785049-G, *LXR *(NR1H2) C-rs1405655-T, T-rs2695121-C and risk of CRC, as well as interactions between genes and consumption of red and processed meat, smoking status and use of NSAID in relation to the development of CRC, in a case-cohort study nested in the prospective population-based Danish Diet, Cancer and Health study.

## Methods

### Studied Subjects

The subjects were selected from the Danish Diet, Cancer and Health study, an ongoing prospective cohort study [38]. Between December 1993 and May 1997, 160,725 individuals aged 50 to 64 years, born in Denmark, living in the Copenhagen and Aarhus areas and having no previous cancers at the time of invitation, were invited to participate in the study. A total of 57,053 persons accepted the invitation.

The colorectal cancer study group has been described previously [17,19,20,39,40].

**Figure 1:**
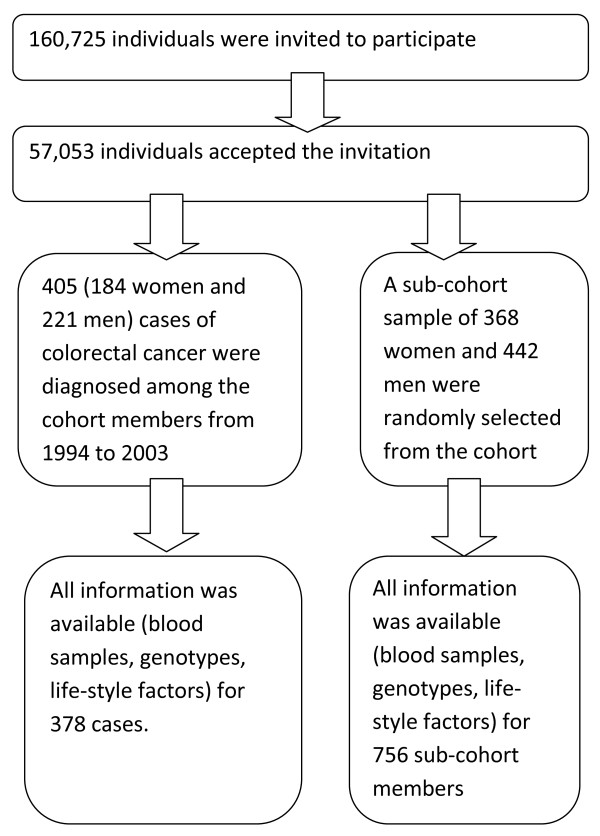
**A flow chart showing the selection of study subjects**. Between December 1993 and May 1997, 160,725 individuals aged 50 to 64 years, born in Denmark, living in the Copenhagen and Aarhus areas and having no previous cancers at the time of invitation, were invited to participate in the study. Please refer to Methods for further details.

In total, 405 cases (184 women and 221 men) of colorectal cancer were diagnosed among the cohort members between 1994 and 2003 and registered in the files of the nationwide Danish Cancer Registry [19]. Within the cohort we defined a sub-cohort sample including 368 women and 442 men who were randomly selected. Cases and the sub-cohort sample were frequency-matched on gender. Blood samples were available for 397 cases and 800 sub-cohort members. The numbers of unknown genotypes were 16 and 37 and the numbers of unavailable food frequency questionnaire were 11 and 17, among cases and sub-cohort members, respectively. All information on genotypes and lifestyle factors was available for 378 cases and 756 sub-cohort members who were included in the statistical analyses. A flow chart is shown in Figure 1.

### Lifestyle variables

At enrolment, detailed information on diet, lifestyle, weight, height, medical treatment, environmental exposures, and other socio-economic factors were collected [38]. In the food-frequency questionnaire, meat consumption was assessed in 12 categories of predefined responses, ranking from 'never' to 'eight times or more per day'. The daily intake was then calculated by using FoodCalc [41], a program which uses population specific standardized recipes and portion sizes. Intake of red meat in grams per day was calculated by adding up intake of beef, veal, pork, lamb and offal. Intake of processed meat in grams per day was calculated by adding up intake of processed red meat, including bacon, smoked ham, salami, frankfurter, Cumberland sausage, cold cuts and liver pâté. Total dietary fibers are calculated by the AOAC methods [42]. Pearson correlation coefficients (adjusted for total energy intake) illustrating the comparison of nutrient scores estimated from the food-frequency questionnaire and from the diet records were 0.39 and 0.53 for dietary fibers, 0.56 and 0.48 for iron, and 0.37 and 0.14 for meat for men and women, respectively [43,44].

Smoking status was categorized in three categories, never smoker, past smoker and current smoker based on the questionnaire.

The lifestyle questionnaire included questions regarding use of specific types of NSAID [45]. Based on all records, we classified study subjects according to use of "any NSAID" (≥ 2 pills per month during one year) at baseline.

Data on hormone replacement therapy (HRT), alcohol intake and anthropometric measurements, and physical activity was obtained from the questionnaire as previously described [38]. The body mass index (BMI) was calculated as weight (kg) per height (m) squared.

### Blood sampling and storage

Blood was collected at enrolment and prepared as previously described [46]. In short, a total of 30 ml blood was collected in citrated (2 × 10 ml) and plain (1 × 10 ml) Venojects from each non-fasting participant, and lymphocytes were isolated and frozen at -20°C within 2 hours. At the end of the day of collection, all samples were stored in liquid nitrogen, at -150 ˚C.

### Genotyping

DNA was isolated from frozen lymphocytes as described [47]. Generally, 100 μg DNA were obtained from 10^7 ^lymphocytes.

Genotypes were determined by Taqman allelic discrimination (ABI 7500/7900HT, Applied Biosystems). Twenty ng of DNA was analysed in 4 μl volumes. Cases and sub-cohort members were mixed during genotyping and laboratory staff was blinded to case/control status during analysis. Triplicates of genotype controls of all three genotypes were included in each run. The controls were selected in pilot genotype determinations of DNA from the same cohort. Ten % of the samples were randomly selected and genotyped again for reproducibility with 100% identity.

NFKB1 ATTG ins/del (rs28362491) was determined as described [48]. Four μl reactions contained ca. 20 ng DNA, 2 μl mastermix (Applied Biosystems, Birkerød, Denmark), 100 nM of each probe and 900 nM primers. Primer sequences were: F: 5'-CTA TGG ACC GCA TGA CTC TAT CAG-3' R: 5'-GGG CTC TGG CTT CCT AGC A-3'. Probe sequences were: NFKB -INS: 5'-FAM-ACC ATT GAT TGG GCC CGG-BHQ-3', NFKB-del: 5'-Yakima Yellow-CCG ACC ATT GGG CCC G-BHQ-3'.

*PXR (NR1I2) *A-24381C (rs1523127), C8055T (rs2276707), A7635G (rs6785049), *LXR-β (NR1H3) *C-rs1405655T, and T-rs2695121C were assayed using pre-developed assays (Applied Biosystems).

### Statistical Analysis

The analyses were performed according to the principles for the analysis of case-cohort studies as described by Barlow [49]. The analyses were performed unweighted. Age was used as the time scale in the Cox regression model. Tests and confidence intervals were based on Wald's test using the robust estimate of the variance-covariance matrix for the regression parameters in the Cox regression model [50].

All models were adjusted for baseline values of established risk factors for colorectal cancer such as BMI (kg/m^2^, continuous), NSAID (yes/no), use of HRT (never/past/current, among women), smoking status (never/past/current), and intake of dietary fibers (g/day, continuous), red meat (g/day, continuous), alcohol intake (g/day, continuous), and physical activity (hours/day, continuous).

We investigated possible interactions between the genes and selected environmental factors using the likelihood ratio test. Tertiles of meat intake was defined as bounderies of intake of red and processed meat among the case group when devided into tertiles. Trend test were calculated using the Wald test. The procedure PHREG in SAS (release 9.1; SAS Institute Inc., Cary, NC, USA) was used for the statistical analyses.

### Ethics

All participants gave verbal and written informed consent. Diet, Cancer and Health and the present sub-study were approved by the regional Ethics Committees on Human Studies in Copenhagen and Aarhus (Jr.nr. (KF)11-037/01 and jr.nr. (KF)01-045/93), and by the Danish Data Protection Agency.

## Results

Characteristics of the study population and risk factors for CRC are shown in Table 1. The genotype distributions among the participants in the sub-cohort sample did not deviate from Hardy-Weinberg equilibrium (results not shown).

**Table 1:** Baseline characteristics of study participants selected from the Danish Diet, Cancer and Health prospective cohort study.

	Cases		Sub-cohort	
	No. (%)	Median (5-95%)	No. (%)	Median (5-95%)
***Total***	378 (100)		756 (100)	
***Gender***				
Men	207 (55)		409 (54)	
Women	171 (45)		347 (46)	
*Age at inclusion*		58 (51-64)		56 (50-64)
*Topology*				
Proximal segment of colon	42 (11)			
Distal segment of colon	148 (39)			
Rectal	136 (36)			
Not specified	52 (14)			
*BMI*		26 (21-34)		26 (21-33)
*Food intake*				
Alcohol, g/day		14 (1-69)		13 (1-61)
Red meat, g/day		82 (37-171)		82 (32-177)
Processed meat, g/day		26 (6-79)		25 (4-76)
Dietary fibres g/day		20 (10-32)		21 (11-34)
*Smoking status at inclusion*				
Never	116 (31)		250 (33)	
Former	113 (30)		238 (31)	
Present	149 (39)		268 (35)	
*NSAID use*				
No	123 (33)		234 (31)	
Yes	255 (67)		522 (69)	
*HRT use among women*				
Never	100 (58)		186 (54)	
Former	26 (15)		58 (17)	
Present	45 (26)		103 (30)	

Observed median values (5-95 percentiles) or percents of the distribution of baseline characteristics among cases and members of the comparison group.

Carriers of the *NFkB *-94del were at 1.45-fold (95% confidence interval (CI): 1.10-1.92) higher risk of CRC than homozygous carriers of the ins allele (Table 2) whereas *PXR *and *LXR *genotypes were not associated with CRC risk (Table 2). Haplotype analyses of the PXR gene showed eight different haplotypes. The most frequent haplotypes were the A-rs1523127-C/A- rs6785049-G/C-rs2276707-T combinations showing CGC, AAC, and the AGC which had frequencies of 0.12, 0.40 and 0.14, respectively. Haplotype analyses of the LXR gene showed four different haplotypes. The frequencies were for the C-rs1405655-T/T-rs2695121-C combinations TC, TT, CT and CC 0.33, 0.30, 0.24, and 0.12, respectively. No association was found between any of the eight *PXR *or four *LXR *haplotypes and CRC (data not shown).

**Table 2:** Incidence rate ratios (IRR) for colorectal cancer for the studied gene polymorphisms.

	**N**_**cases**_	**N**_**sub-cohort**_	**IRR**^**A**^	95% CI	**IRR**^**B**^	95% CI	**p-value**^**C**^
NFkB -94ins/del							
II	121	307	1.00	-	1.00	-	0.03
ID	195	347	1.46	(1.10-1.93)	1.44	(1.07-1.92)	
DD	62	102	1.50	(1.02-2.22)	1.50	(1.00-2.25)	
ID and DD	257	449	1.47	(1.12-1.92)	1.45	(1.10-1.92)	
PXR rs1523127							
AA	127	257	1.00	-	1.00	-	0.78
AC	188	371	0.99	(0.74-1.31)	0.99	(0.74-1.32)	
CC	63	128	1.08	(0.74-1.57)	1.12	(0.77-1.65)	
AC and CC	251	499	1.01	(0.77-1.32)	1.02	(0.77-1.34)	
PXR rs2276707							
CC	237	441	1.00	-	1.00	-	0.66
CT	122	270	0.87	(0.67-1.14)	0.88	(0.67-1.17)	
TT	19	45	0.83	(0.46-1.49)	0.89	(0.49-1.62)	
CT and TT	141	315	0.87	(0.67-1.12)	0.88	(0.68-1.15)	
PXR rs6785049							
AA	136	259	1.00	-	1.00	-	0.60
AG	187	398	0.91	(0.69-1.21)	0.90	(0.67-1.21)	
GG	55	99	1.10	(0.74-1.64)	1.09	(0.72-1.65)	
AG and GG	242	497	0.95	(0.73-1.24)	0.94	(0.71-1.24)	
LXR rs1405655							
CC	169	347	1.00	-	1.00	-	0.88
CT	169	335	1.00	(0.77-1.31)	1.00	(0.76-1.31)	
TT	40	74	1.05	(0.68-1.63)	1.12	(0.71-1.76)	
CT and TT	209	409	1.01	(0.79-1.31)	1.02	(0.79-1.32)	
LXR rs2695121							
TT	117	221	1.00	-	1.00	-	0.36
CT	187	368	0.93	(0.69-1.25)	0.91	(0.67-1.23)	
CC	74	167	0.78	(0.54-1.12)	0.77	(0.53-1.11)	
CT and CC	261	535	0.88	(0.67-1.17)	0.86	(0.65-1.15)	

^A^Crude (adjusted for age and sex).

^B^Additionally adjusted for status of HRT (women only), smoking status, alcohol, dietary fibre, red meat, physical activity and BMI and NSAID use.

^C^p-value for trend for the adjusted estimates.

Since we observed no allele-dose effects, all variant allele carriers were combined in subsequent analyses to maximize the statistical power. Del-allele carriers of *NFkB *-94ins/del were at 3% higher risk pr 25 g meat/day (CI: 0.98-1.09) whereas among homozygous carriers of the ins-allele, the association was in the opposite direction (IRR pr 25 g/day: 0.97, CI: 0.90-1.04, p for interaction= 0.03) (Table 3). Table 4 shows the IRRs for tertiles of intake of red and processed meat subdivided by the *NFkB *genotypes. Among homozygous ins-allele carriers of the *NFkB *-94ins/del polymorphism, IRRs were unchanged across tertiles of meat intake, whereas for del-allele carriers, the risk of CRC was higher for the second and the third tertile of meat intake compared to the first tertile (Table 4). In this analysis, there was no statistical significant interaction.

**Table 3:** Incidence rate ratios (IRR) for colorectal cancer per additional intake of 25 g red or processed meat subdivided by the studied genotypes.

			*Red and processed meat*				
	**N**_**cases**_	**N**_**sub-cohort**_	**IRR**^**A**^	95% CI	**IRR**^**B**^	95% CI	**p-value**^**C**^
NFkB -94ins/del							
II	121	307	0.96	(0.90-1.03)	0.97	(0.90-1.04)	0.03
ID and DD	257	449	1.03	(0.98-1.08)	1.03	(0.98-1.09)	
PXR rs1523127							
AA	127	257	1.04	(0.98-1.12)	1.04	(0.97-1.12)	0.28
AC and CC	251	499	1.01	(0.96-1.06)	1.01	(0.96-1.07)	
PXR rs2276707							
CC	237	441	1.02	(0.97-1.08)	1.02	(0.97-1.08)	0.53
CT and TT	141	315	1.01	(0.95-1.07)	1.01	(0.94-1.08)	
PXR rs6785049							
AA	136	259	1.01	(0.95-1.07)	1.01	(0.95-1.08)	0.72
AG and GG	242	497	1.02	(0.97-1.08)	1.02	(0.97-1.08)	
LXR rs1405655							
CC	169	347	1.02	(0.97-1.07)	1.02	(0.96-1.08)	0.91
CT and TT	209	409	1.02	(0.96-1.08)	1.02	(0.96-1.08)	
LXR rs2695121							
TT	117	221	1.04	(0.98-1.12)	1.05	(0.97-1.12)	0.28
CT and CC	261	535	1.01	(0.96-1.07)	1.01	(0.96-1.07)	

^A^Crude (adjusted for age and sex).

^B^Additionally adjusted for status of HRT (women only), smoking status, alcohol, dietary fibre, physical activity, NSAID use and BMI.

^C^p-value for interaction between genotype and red and processed meat for the adjusted estimates.

**Table 4:** Incidence rate ratios (IRR) for colorectal cancer for tertiles of intake of red and processed meat subdivided by the *NFkB *genotypes.

	*Red and processed meat*						**IRR**^**A **^**95% CI**						**p-value**^**B**^
	**1. tertile**		**2. tertile**		**3. tertile**		***Red and processed meat***						

	**N**_**C**_	**N**_**S**_	**N**_**C**_	**N**_**S**_	**N**_**C**_	**N**_**S**_	**<91.4 g/day**		**91.4-130.2 g/day**		**>130.2 g/day**		

NFkB -94ins/del													

II	46	107	35	98	40	102	1.00	-	0.88	(0.50-1.54)	1.07	(0.61-1.88)	0.16

ID and DD	78	157	91	137	88	155	1.19	(0.75-1.91)	1.70	(1.06-2.74)	1.44	(0.89-2.34)	

^A^Adjusted for status of HRT (women only), smoking status, alcohol, dietary fibre, BMI, physical activity and NSAID use.

^B^p-value for interaction.

No interactions between smoking status or NSAID use and the studied genotypes were found (Tables 5 and 6).

**Table 5:** Interaction between the studied polymorphisms and smoking status.

	Smoking						**IRR**^**A **^**95% CI**						**p-value**^**B**^
	Never		Past		Current		Smoking						
	**N**_**C**_	**N**_**S**_	**N**_**C**_	**N**_**S**_	**N**_**C**_	**N**_**S**_	Never		Past		Current		
NFkB -94ins/del													
II	37	101	37	101	47	105	1.00	-	0.89	(0.51-1.55)	1.15	(0.67-1.97)	0.84
ID and DD	79	149	76	137	102	163	1.45	(0.89-2.37)	1.41	(0.86-2.33)	1.55	(0.96-2.51)	
PXR rs1523127													
AA	33	94	42	76	52	87	1.00	-	1.37	(0.77-2.47)	1.55	(0.89-2.70)	0.08
AC and CC	83	156	71	162	97	181	1.49	(0.90-2.45)	1.15	(0.69-1.91)	1.38	(0.84-2.28)	
PXR rs2276707													
CC	63	141	70	135	104	165	1.00	-	1.01	(0.65-1.57)	1.24	(0.82-1.87)	0.43
CT and TT	53	109	43	103	45	103	1.06	(0.66-1.68)	0.89	(0.54-1.47)	0.93	(0.57-1.51)	
PXR rs6785049													
AA	40	89	38	85	58	85	1.00	-	0.92	(0.52-1.61)	1.37	(0.80-2.33)	0.28
AG and GG	76	161	75	153	91	183	1.06	(0.65-1.72)	1.00	(0.61-1.63)	1.04	(0.64-1.67)	
LXR rs1405655													
CC	49	117	54	116	66	114	1.00	-	0.93	(0.56-1.54)	1.18	(0.72-1.92)	0.83
CT and TT	67	133	59	122	83	154	1.07	(0.66-1.72)	1.01	(0.62-1.64)	1.11	(0.71-1.74)	
LXR rs2695121													
TT	36	74	35	63	46	84	1.00	-	1.12	(0.61-2.07)	1.20	(0.67-2.13)	0.67
CT and CC	80	176	78	175	103	184	0.98	(0.59-1.63)	0.85	(0.51-1.42)	1.03	(0.63-1.70)	

^A^Adjusted for age, sex, status of hormone replacement therapy (HRT)(women only), alcohol, dietary fibre and BMI, physical activity and NSAID use.

^B ^P-value for interaction.

**Table 6:** Interaction between gene polymorphisms and NSAID use.

	NSAID				**IRR**^**A **^**95% CI**				**IRR**^**B **^**95% CI**				**p-value**^**C**^
	NO		YES		NSAID				NSAID				
	**N**_**C**_	**N**_**S**_	**N**_**C**_	**N**_**S**_	NO		YES		NO		YES		
NFkB -94ins/del													
II	83	210	38	97	1.00	-	1.09	(0.69-1.73)	1.00	-	1.03	(0.64-1.65)	0.82
ID and DD	172	312	85	137	1.47	(1.06-2.03)	1.60	(1.09-2.35)	1.43	(1.02-1.99)	1.55	(1.04-2.31)	
PXR rs1523127													
AA	83	172	44	85	1.00	-	1.09	(0.69-1.73)	1.00	-	1.07	(0.67-1.71)	0.99
AC and CC	172	350	79	149	1.01	(0.73-1.40)	1.11	(0.75-1.63)	1.02	(0.73-1.43)	1.09	(0.73-1.63)	
PXR rs2276707													
CC	161	296	76	145	1.00	-	1.01	(0.72-1.43)	1.00	-	0.96	(0.67-1.37)	0.25
CT and TT	94	226	47	89	0.81	(0.59-1.12)	1.01	(0.67-1.53)	0.81	(0.59-1.12)	1.02	(0.66-1.56)	
PXR rs6785049													
AA	90	180	46	79	1.00	-	1.11	(0.70-1.74)	1.00	-	1.07	(0.67-1.71)	0.98
AG and GG	165	342	77	155	0.96	(0.69-1.32)	1.04	(0.71-1.53)	0.94	(0.67-1.32)	1.00	(0.67-1.49)	
LXR rs1405655													
CC	117	238	52	109	1.00	-	1.00	(0.67-1.50)	1.00	-	0.94	(0.62-1.44)	0.32
CT and TT	138	284	71	125	0.96	(0.71-1.31)	1.13	(0.78-1.65)	0.95	(0.69-1.30)	1.12	(0.76-1.64)	
LXR rs2695121													
TT	85	161	32	60	1.00	-	1.08	(0.64-1.80)	1.00	-	1.02	(0.58-1.78)	0.75
CT and CC	170	361	91	174	0.87	(0.62-1.21)	0.97	(0.67-1.41)	0.84	(0.60-1.19)	0.93	(0.63-1.36)	

^A^Crude (adjusted for age and sex)

^B^Additionally adjusted for status of hormone replacement therapy (HRT), smoking status, alcohol, dietary fibre, red meat, physical activity and BMI.

^C ^P-value for interaction for the adjusted estimates.

## Discussion

Carriers of the del-allele of *NFkB *-94ins/del polymorphism were at statistically significantly higher risk of CRC than the homozygous carriers of the ins-allele.

Prospective studies have the advantage in relation to examining gene-environmental interactions that they are not encumbered by recall bias. In the present study, cases and cohort sample were selected from the same cohort, which together with complete follow up of the participants minimised the risk of selection bias. We have used the case-cohort study design in the present study. The case-cohort analysis is a more flexible design than a nested case-control design [49]. It permits us to use one large, randomly selected comparison group as comparison group for several nested studies within the Diet, Cancer and Health cohort, saving resources and valuable DNA, and the obtained risk estimates can be related directly to the cohort, which is population-based. We have previously used the same design in other studies of colorectal cancer [17,19,20,39,51], lung cancer [52-55], and coronary heart disease [56-59].

A main strength of our study is a well characterized study population. Information on lifestyle factors were collected at enrolment for all participants which minimised the risk of differential misclassification of cases and comparison group. However, lifestyle factors were only collected once, and may thus not be representative for the lifestyle during follow-up. This is, however, not expected to result in differential misclassification. Furthermore, information on food intake was based on a semi-quantitatively food frequency questionnaire [38,44], which was evaluated and found usable [43]. The results were adjusted for known confounding factors affecting the risk of CRC in this cohort including dietary factors, body mass index (BMI), alcohol, smoking status, physical activity and NSAID use [1]. An additional strength of the present study is the high exposure of red and processed meat and tobacco smoke in the present study [3,9]. Limitations of the study include the relatively small sample size. Therefore, heterozygous and homozygous variant genotype carriers were combined for the analyses of interactions to obtain sufficient statistical power. Nevertheless, in the light of the obtained P-values and the number of statistical testes performed, we cannot exclude that our positive findings may be due to chance.

We found a weak interaction between *NFkB *-94ins/del polymorphism and intake of red and processed meat in relation to CRC risk (Table 3). In the interaction analysis, we calculated the slopes of two curves describing the relationship between meat intake and CRC risk for homozygous carriers of the ins-allele and for carriers of the del-allele, respectively. However, either of the slopes is statistically significantly different from 1, as indicted by the confidence intervals. However, the two slopes were statistically significantly different from each other as indicated by the p-value for the interaction (p = 0.03). Furthermore, among homozygous carriers of the ins-allele of the *NFkB *-94ins/del polymorphism, risk of CRC was unchanged across tertiles of meat intake, whereas for del-allele carriers, the risk of CRC was higher for the second and the third tertile of meat intake compared to the first tertile (Table 4). In this analysis, there was no statistically significant interaction. Our study thus suggests that the *NFkB *polymorphism may be most important in study populations with high consumption of red and processed meat. The *NFkB *-94ins/del del-allele was associated with risk of CRC among Swedes, whereas no association was found among Chinese [32]. Hence, the difference between the findings in the Danish and Swedish on one hand and the Chinese on the other may be related to differences in meat exposure. Meat intake is much high in high income countries, including Denmark and Sweden, compared to low income countries, including China, respectively [3].

We have previously found interaction between meat intake and polymorphisms in the xenobiotic transporter *MDR1 *in relation to CRC in this study group [20]. Combined carriers of *MDR1 *intron 3 G-rs3789243A A-allele and *NFkB *-94ins/del del-allele have an IRR of 1.85 (95% confidence interval (CI): 1.15-3.00, p = 0.007) compared to the homozygous G-allele and ins-allele carriers, suggesting additive but no synergistic effect by having both risk alleles. However, the statistical power to explore gene-gene interactions was low. The two studies may suggest that a lower NFkB response by the *NFkB *del-allele carriers after stimulation by intestinal carcinogens may lead to a lower expression of the *MDR1 *[21] and, thus, a lower transport activity and, eventually, an impaired defence against intestinal uptake of luminal carcinogens compared to homozygous ins-allele carriers. Furthermore, interaction between meat and *NFkB *may be caused by a lower activation of *NFkB *del-allele relative to the ins-allele resulting in a high load of reactive oxygen species provided by heme degradation and which may contribute to carcinogenesis [60].

We found no interaction between the *NFkB *-94ins/del polymorphism and smoking status, suggesting that the carcinogen exposure from tobacco smoke, which comes from ingestion of inhaled tobacco smoke particles, is less than the carcinogen exposure from ingestion of red and processed meat. This is in accordance with our previous finding of smoking being a weaker risk factor for CRC than red and processed meat [20].

We found no statistically significant associations between the *PXR *and *LXR *polymorphisms and CRC risk, and no interaction with smoking status, use of NSAID, or intake of red and processed meat. These two genes have not previously been investigated in relation to CRC. We had from 73% to 99% chance to detect a dominant effect of the three studied polymorphisms in *PXR *using the ORs from the study of Dring [61] of 1.3-2.9 and similarly more than 84% chance of detecting a dominant effect with an OR of 1.5 in *LXR*. Our findings do therefore not support important roles of *PXR *and *LXR *in CRC etiology. This is in accordance with the observation that *NFkB *activation suppress the action of *PXR *and vice versa [62,63].

We found no interaction between NSAID use and *NFκB, PXR *and *LXR *genotypes. A risk reducing affect of NSAID may be dependent on functional NFκB, as aspirin has been shown to induce apoptosis in intestinal cell lines by a NFκB dependent mechanism [64]. Long-term use of NSAID was associated with a protective effect against CRC in the "Diet, Cancer and Health" cohort, used in the present study [45]. Hence, the results do not support important roles of the studied genotypes in interaction with NSAID in CRC aetiology.

## Conclusions

The present study indicated that the ins/del *NFkB *polymorphism may be involved in CRC etiologi. The ins/del *NFkB *polymorphism may affect the risk of CRC by exposure of meat as carriers of the *NFkB *del-allele were more susceptible to meat carcinogens than homozygous ins-allele carriers. This is the first study finding interaction between *NFkB *and meat intake in relation to CRC. However, the findings must be further evaluated in large populations with high meat intake.

## Abbreviations

ABCB2: ATP-binding cassette (ABC) transporter B2 (MDR1); ABCC2: ATP-binding cassette (ABC) transporter C2 (MRP2); ABCG2: ATP-binding cassette (ABC) transporter B2 (BCRP); COX-2: cyclooxygenase-2; CRC: colorectal cancer; IL-1β: interleukin 1-β; LXR: Liver X receptor (NR1H3); MMP-9: matrix metalloproteinase-9; LPS: lipopolysaccharide; NSAID: non-steroidal anti-inflammatory drugs; NFkB: nuclear factor kappa-B (NFKB1); PXR: pregnane X receptor (NR1I2); SNP: single nucleotide polymorphism; TNF-α: tumor nekrosis factor-α; VEGF: vascular endothelial growth factor;

## Competing interests

The authors declare that they have no competing interests.

## Authors' contributions

UV carried out the molecular genetic studies. UV, KO, AT participated in the design of the study and JC performed the statistical analysis. VA conceived the study and drafted the manuscript. All authors read and approved the final manuscript.

## Pre-publication history

The pre-publication history for this paper can be accessed here:

http://www.biomedcentral.com/1471-2407/10/484/prepub
